# A Wearable Inertial Sensor Approach for Locomotion and Localization Recognition on Physical Activity

**DOI:** 10.3390/s24030735

**Published:** 2024-01-23

**Authors:** Danyal Khan, Naif Al Mudawi, Maha Abdelhaq, Abdulwahab Alazeb, Saud S. Alotaibi, Asaad Algarni, Ahmad Jalal

**Affiliations:** 1 Faculty of Computing ad AI, Air University, E-9, Islamabad 44000, Pakistan; 211651@students.au.edu.pk; 2 Department of Computer Science, College of Computer Science and Information System, Najran University, Najran 55461, Saudi Arabia; 3 Department of Information Technology, College of Computer and Information Sciences, Princess Nourah Bint Abdulrahman University, P.O. Box 84428, Riyadh 11671, Saudi Arabia; 4 Information Systems Department, College of Computer and Information Systems, Umm Al-Qura University, Makkah 24382, Saudi Arabia; 5 Department of Computer Sciences, Faculty of Computing and Information Technology, Northern Border University, Rafha 91911, Saudi Arabia; asaad.algarni@nbu.edu.sa

**Keywords:** human activity recognition, smartphone sensors, synthetic minority oversampling technique (SMOTE), long short-term memory (LSTM)

## Abstract

Advancements in sensing technology have expanded the capabilities of both wearable devices and smartphones, which are now commonly equipped with inertial sensors such as accelerometers and gyroscopes. Initially, these sensors were used for device feature advancement, but now, they can be used for a variety of applications. Human activity recognition (HAR) is an interesting research area that can be used for many applications like health monitoring, sports, fitness, medical purposes, etc. In this research, we designed an advanced system that recognizes different human locomotion and localization activities. The data were collected from raw sensors that contain noise. In the first step, we detail our noise removal process, which employs a Chebyshev type 1 filter to clean the raw sensor data, and then the signal is segmented by utilizing Hamming windows. After that, features were extracted for different sensors. To select the best feature for the system, the recursive feature elimination method was used. We then used SMOTE data augmentation techniques to solve the imbalanced nature of the Extrasensory dataset. Finally, the augmented and balanced data were sent to a long short-term memory (LSTM) deep learning classifier for classification. The datasets used in this research were Real-World Har, Real-Life Har, and Extrasensory. The presented system achieved 89% for Real-Life Har, 85% for Real-World Har, and 95% for the Extrasensory dataset. The proposed system outperforms the available state-of-the-art methods.

## 1. Introduction

Human activity recognition (HAR) has emerged as a pivotal domain in ubiquitous computing, with applications spanning from healthcare monitoring [1,2,3] to advanced user interfaces. At the heart of HAR is the ability to interpret and predict human actions using a myriad of sensors [4,5,6] and data sources. Among these, smartphone and wearable sensors have become particularly significant due to their distinct attributes and widespread presence in everyday devices. Inertial sensors, primarily accelerometers and gyroscopes, are embedded in a multitude of devices, including smartphones, smartwatches, and other wearables. These sensors offer distinct advantages: they can function autonomously without the need for external infrastructure, unlike methods that rely on cameras and require a clear line of sight. Furthermore, their low power consumption makes them ideal for long-term, continuous monitoring applications. On the other hand, GPS data provide valuable spatial and temporal information, crucial for understanding and predicting human movement and behavior in diverse environments [7,8,9,10]. Beyond HAR, both inertial sensors and GPS data find applications in various domains [11,12,13]. For example, gait analysis [14,15] utilizes inertial sensors to analyze a person’s identity based on their unique walking pattern, presenting a novel avenue for biometric authentication. Similarly, GPS data have revolutionized location-based services [16,17,18], urban planning, and transportation studies. Nevertheless, utilizing the full potential of inertial sensors and GPS data for human activity recognition presents challenges. Variability in human movements, noise interference, diverse environments, and the sheer array of possible activities make accurate recognition a complex task. In this study, we have crafted an advanced HAR system that distinguishes between different human locomotion and localization activities using data from smartphones and smartwatch sensors [19,20]. Our methodology is rigorous, involving an effective noise reduction process that uses a Chebyshev Type I filter, followed by signal segmentation with a Hamming window. Feature extraction is then meticulously performed, and the most informative features are selected via recursive feature elimination. Addressing the challenge of imbalanced datasets, we employ SMOTE for data augmentation, leading to a balanced dataset that feeds into an LSTM classifier for final activity recognition.

The main contributions of our work are manifold, including:

- The study applies the synthetic minority oversampling technique (SMOTE) to address class imbalances within the Extrasensory dataset, leading to a significant improvement in the model’s accuracy and robustness across underrepresented activities.
- Innovative feature identification and extraction for localization activities, contributing to the field’s understanding of movement in space.
- A hybrid approach combining various signal processing and machine learning techniques to accurately recognize human activity patterns.
- Comprehensive evaluation using three benchmark datasets, with our system showing superior performance compared to state-of-the-art methods.

The rest of the article is arranged into the following sections. Section 2 presents the literature review in the field of HAR via smartphone sensors. Section 3 explains the proposed system design. Section 4 shows the experimental setup. Section 5 presents the experimental results obtained in this study and also the results’ discussion. Section 6 introduces the implications of the proposal system and Section 7 describes the conclusion and future work.

## 2. Related Work

The field of human locomotion monitoring through remote sensing has made significant strides, yielding increasingly effective methodologies. Our extensive review of the existing literature has provided insights into various remote sensing techniques previously utilized, setting the stage for the creation of a more advanced model to tackle the research issue at hand. Inertial sensors are widely recognized for their pivotal role in human locomotion activity recognition (HLAR). The ubiquity of smartphones, equipped with advanced inertial sensors such as accelerometers, gyroscopes, and magnetometers, has greatly enriched research in remote sensing-based HLAR (RS-HLAR). These embedded sensors provide a wealth of valuable data, facilitating the development of sophisticated models and techniques for accurate and efficient activity tracking and analysis.

The study presented in [21] utilized smartphone-embedded gyroscopes and accelerometers to derive hand-crafted features. They employed neighborhood component analysis to identify the most effective features. Subsequently, these features were input into a dense neural network, which was tasked with classifying various types of locomotion activities.

In their study, Xie et al. [22] evaluated the efficacy of various kernel functions within a support vector machine (SVM) framework to classify different locomotion activities, including ascending and descending stairs, walking, and standing. They utilized data from smartphone-embedded accelerometers, gyroscopes, and magnetometers to distill a range of features from both frequency and time domains. Subsequently, they implemented a multiclass SVM in a one-versus-all scheme. The robustness of their approach was substantiated by a 10-fold cross-validation process, which confirmed the reliability of their classification system. In the research presented in [23], the authors explored a hybrid CNN-LSTM architecture that incorporated an attention mechanism to enhance the model’s focus on relevant features for activity classification. This system utilized data from smartphone sensors, specifically accelerometers, gyroscopes, and magnetometers, to monitor human motion. Another study [24] proposed a combination model consisting of deep bidirectional long short-term memory (DBLSTM) and CNN. The DBLSTM component serialized sensor data, providing bidirectional temporal context, while the CNN excelled at feature extraction, an area where DBLSTM alone might falter. The culmination of this process involved a softmax layer, which was responsible for the final classification of the activities. In the study conducted by Hsu et al. [25], the researchers employed wearable inertial sensors to monitor accelerations and angular velocities of the human body. These sensors, one affixed to the wrist and the other to the ankle, transmitted data wirelessly to a computer for analysis. The computer system processed these data using a sophisticated algorithm that combined nonparametric weighted feature extraction with principal component analysis to differentiate between various human activities. The approach had certain limitations, primarily the use of only two sensors, which may not fully capture the complexity of human movements and postures. Additionally, the reliance on a wireless connection for data transmission introduced potential issues of reliability and accessibility in certain settings. The study in [26] described a system utilizing accelerometer data to discern human activity by categorizing features into three distinct groups: motion, orientation, and rotation. It analyzes how each set of features, as well as their combinations, contributes to the accuracy of activity recognition. To classify activities, the system employs various machine learning algorithms, such as decision trees, naive Bayes classifiers, and random forests. A notable aspect of the system is its reliance on an accelerometer-based approach for recognizing activities and its systematic organization of features. However, the dataset’s limited scope, collected from only ten individuals, raises questions about the system’s applicability to a broader population. Furthermore, while the use of conventional machine learning classifiers provides a baseline for recognition tasks, there is potential for improvement by adopting more sophisticated models. In comparison, our approach uses a more expansive dataset, the Extrasensory dataset, which includes data from 60 participants, enhancing the model’s generalizability. This system also employs a hybrid LSTM, an advanced classification technique that combines the strengths of both traditional machine learning and deep learning. The use of hybrid LSTM and a more extensive dataset allows the system to achieve state-of-the-art results.

Chetty et al. [27] presented an advanced data analysis technique for the recognition of human activity using smartphone inertial sensors. Their approach utilized a suite of machine learning classifiers, including random forests, ensemble learning, and lazy learning. These classifiers were informed by an information theory-based feature ranking algorithm, which was crucial for selecting the most relevant features. The system’s reliance on smartphone sensors for activity detection underscores its modernity and relevance. The incorporation of an information theory framework for feature ranking highlights an effort to base feature selection on solid mathematical principles. One limitation of their system, however, was its training on a single dataset. This could potentially restrict the system’s ability to perform optimally across various real-world scenarios, as a single dataset may not capture the complexity and variability of human activities in different environments. By contrast, the proposed system is trained on three benchmark datasets, including the Extrasensory dataset, which is known for its diverse range of activities and its collection in uncontrolled, ‘in the wild’ conditions. This lack of constraints on the participants during data collection contributes to the robustness and reliability of the system, potentially making it more adaptable and dependable for real-world applications compared to the single-dataset approach of Chetty et al. This breadth of data underpinning the proposed system promises enhanced performance in various settings, affirming its suitability for widespread deployment. Ehatisham-ul-Haq et al. [28] developed a comprehensive framework for recognizing human contexts through a novel activity-aware method. The framework predicts user contexts by recognizing physical activities (PAR) and learning patterns from a variety of behavioral situations. It correlates five daily activities—lying, sitting, standing, walking, and running—with fourteen different behavioral contexts, including various phone positions. For its evaluation, the framework utilized random forests alongside other machine learning classifiers. The system excels at using human activity recognition to deduce the context of an activity and incorporates additional data, such as the subject’s location and concurrent secondary activities, like walking while eating or sitting and talking. Its primary reliance on accelerometer data, however, suggests potential enhancements. Incorporating GPS and microphone data could vastly improve location estimation and the detection of nuanced activities. In comparison, the proposed system spans a more extensive array of sensors: a smartphone accelerometer, magnetometer, and gyroscope; a smartwatch accelerometer and compass; as well as a smartphone GPS and microphone. This multisensor integration enhances the system’s robustness in activity recognition and location determination. Furthermore, it employs hybrid long short-term memory (LSTM), an amalgamation of machine learning and deep learning techniques, offering superior performance over the simpler random forest classifier used by Ehatisham-ul-Haq et al., especially in classifying complex activities with higher accuracy. Mutegeki et al. [29] introduced a straightforward approach for human locomotion recognition (HLR) by utilizing a combination of convolutional neural networks (CNN) and long short-term memory (LSTM) networks. Opting to use these networks, they bypassed the need for a feature engineering module, aiming to enhance system performance for a limited set of activities. Various configurations of CNN and LSTM layers have been explored in research; however, the findings in [29] suggest that such a dual-network model exhibited diminished effectiveness when it came to more complicated, atomic-level actions. Moreover, as the complexity of the model increased, there was a noticeable rise in softmax loss, indicating that the integration of CNN and LSTM layers did not necessarily lead to improved outcomes. In contrast, the proposed system is capable of differentiating complicated and varying activities efficiently. Hajjej et al. [30] employed a methodology centered around data from a solitary sensor and utilized a Quaternion-based filtering technique. Following this, they applied several segmentation methods to create data windows. Subsequently, patterns were identified, and relevant features were extracted and chosen for further analysis. For the classification phase, an LSTM network was utilized. Despite the advancements, the study encountered certain limitations such as delays in response time and elevated computational demands. The study presented in [31] developed a methodology for recognizing human activities through wearable devices, utilizing IMU sensors to capture the data from executed actions. The data underwent a multistep preprocessing routine that included the elimination of dropouts, application of global normalization, moving average, and the use of sliding overlapping windows along with segmentation techniques. To classify the different human activities, the study utilized five distinct deep learning architectures, namely CNN, RNN, LSTM, BiLSTM, and GRU. Nonetheless, the system’s temporal efficiency was less than optimal, reflected in the extensive training time across 892,839 learning epochs. In comparison, the proposed system achieved a very good accuracy rate across all datasets with less training time across 50 epochs. In [32], the authors apply machine learning techniques to analyze clinical EEG data for human activity recognition (HAR). Key EEG features like delta, theta, alpha, beta, and gamma waveforms are extracted using the Welch periodogram and fast Fourier transforms. These features are then used to train advanced machine learning classifiers like random forest, gradient boosting, and extreme gradient boosting. One of the study’s primary limitations is its reliance on clinical-grade EEG data, which may not be as applicable in consumer-grade or real-world scenarios. Additionally, the research is based on a relatively homogeneous group of participants, potentially limiting the generalizability of the findings across diverse populations. The authors in [33] apply various machine learning algorithms to improve human activity recognition (HAR) using smartphone sensor data. They use a real-life oriented dataset, which includes accelerometer, gyroscope, magnetometer, and GPS data. Their approach involves extensive data preprocessing and feature extraction, where they test a range of sliding window sizes from 20 to 90 s. The researchers explore different algorithms, including tree-based models like random forests and extreme gradient boosting, along with support vector machines, multilayer perceptrons, and others. They perform a grid search for optimal hyperparameter combinations and validate the models using stratified k-folding and hold-out techniques. The study acknowledges limitations in their feature set, noting that the proposed additions did not significantly enhance model performance compared to the primary set. Another limitation is the difficulty in optimally discerning the ‘active’ activity category, which often gets confused with the ‘inactive’ or ‘walking’ categories. This indicates a need for more nuanced feature engineering or the adoption of advanced algorithms for better classification of complex activities.

## 3. Proposed System Methodology

**Figure 1:**
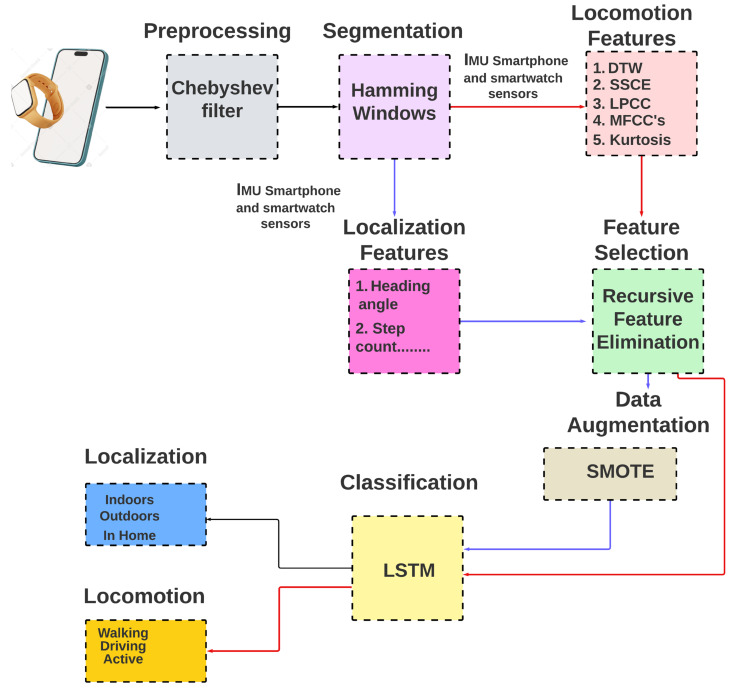
The proposed system architecture.

The data for our study were sourced from three prominent datasets: Real-World HAR, Real-Life HAR, and Extrasensory. In the first stage, we removed the unwanted data using the Chebyshev 1 filter [32]. Then, Hamming windowing and segmentation technique was applied. In the third step, we extracted different features for GPS, IMU, and microphone sensors. To select the best feature vector for the system, we used the recursive feature elimination feature selection [33,34] technique. We observed that the Extrasensory dataset has imbalanced data for some activities; we employed the synthetic minority oversampling technique (SMOTE) to address this imbalance, particularly targeting the ‘at school’ and ‘outdoors’ classes. By doing so, we aimed to enhance the model’s ability to recognize these underrepresented activities without being biased toward the majority classes. Then, the data were sent to the long short-term memory (LSTM). The methodology of the proposed system is shown in Figure 1.

### 3.1. Signal Denoising

**Figure 2:**
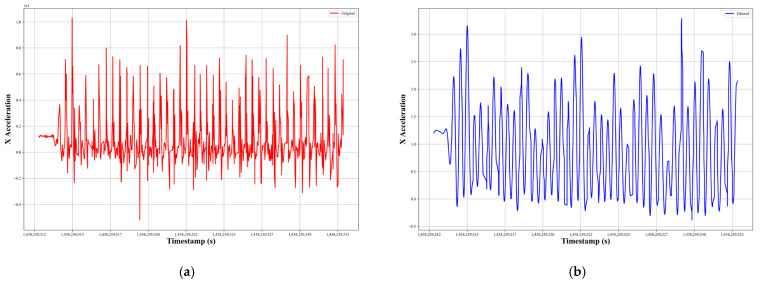
(**a**) Noisy and (**b**) filtered signal for accelerometer sensor.

The preprocessing of sensor data using a Chebyshev Type I filter [35] is a critical step in our methodology to ensure that the signals are optimized for the subsequent feature extraction phase. We applied a passband ripple of 0.5 dB (rp). The Chebyshev Type I filter is characterized by its ripple in the passband and a monotonic (non-oscillatory) roll-off in the stopband. The filtering was applied separately to each channel of the sensor data, ensuring that each signal was smoothed and ready for feature extraction. Mathematically, the magnitude response of the Chebyshev Type I filter is defined by the equation

(1) $$\left|H(w)\right|=\frac{1}{\sqrt{1+\in^{2}T_{n}^{2}\left(\frac{\omega }{\omega_{0}}\right)}}$$

where H(w) is the gain of the filter at frequency ω*,* ∈ is the ripple factor, $T_{n}$ is the Chebyshev polynomial of the first kind of order *n*, and $\omega_{0}$ is the cutoff frequency of the filter. Figure 2 presents a comparative visualization of the accelerometer sensor data before and after the application of the filter. On the left side of the figure, the original noisy signal is displayed, characterized by fluctuations that could potentially obscure the true pattern of movement. On the right side, we show the filtered signal, where the effect of the Chebyshev Type I filter is evident: the noise is significantly reduced, and the signal’s essential features are more pronounced. The relevance of these results to the overall system is twofold. Firstly, they validate the effectiveness of the Chebyshev Type I filter in enhancing the signal quality by attenuating high-frequency noise, which could interfere with accurate activity recognition. Secondly, the filtered signals provide a clearer basis for feature extraction, which is important for the accurate classification of human activities. By improving the signal quality, we aim to increase the reliability of the features extracted, and by extension, the performance of the proposed model employed in the study.

### 3.2. Signal Windowing and Segmentation

We employed the Hamming window technique [36,37] to segment the continuous sensor data stream into overlapping windows. We selected a window size of 100 samples to create overlapping segments from the continuous sensor data stream. This window size was chosen based on a trade-off between capturing sufficient information to represent the characteristics of different activities and computational efficiency. A smaller window size might not encapsulate the complete representation of the activity, while a larger window size may lead to increased computational cost and possibly incorporate transitions between different activities. Additionally, a 50% overlap between consecutive windows resulted in a step size of 50 samples for advancing the window through the data stream. This overlap is intended to ensure that we do not miss any transitional states between activities and to maintain the continuity and smoothness of the signal across window boundaries. The 50% overlap is a common practice in windowing techniques, balancing the need for capturing sufficient variations in the signal with the computational cost associated with processing overlapping segments.

**Figure 3:**
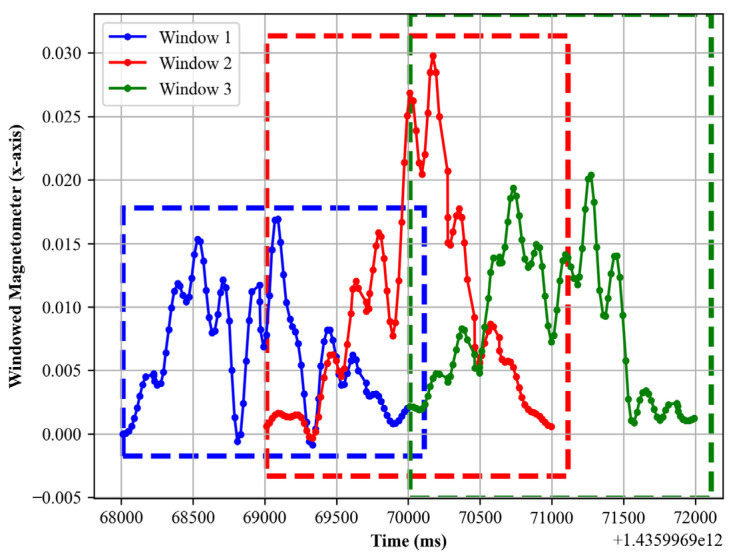
Hamming window’s first 3 windows for magnetometer data.

The Hamming window is defined mathematically by the equation

(2) $$w\left(x\right)=0.54-0.46cos\left(\frac{2\pi x}{X-1}\right)$$

where $w\left(x\right)$ is the window function, X is the window length, and *x* is the sample index, 0≤x≤X−1. The plot is presented in Figure 3.

### 3.3. Feature Extraction

In this section, we listed all the feature lists used in the study specifically aligned with each type of sensor data. Table 1 presents the list of features along with sensor data.

#### 3.3.1. Feature Extraction for Locomotion Activity

Feature extraction is a crucial step in machine learning, as it involves selecting and transforming relevant characteristics of the raw data into a suitable representation for training models. In this study, we divided the feature extraction [38,39,40,41] phase into two parts: in the first part, we extract features for locomotion activity sensors, and in the second phase, localization activity data are processed separately and different features are extracted.

##### Linear Prediction Cepstral Coefficients (LPCC)

We applied a methodical approach to compute the linear prediction cepstral coefficients (LPCC) [42,43] for various locomotion activities. Initially, we extracted the relevant motion signal data. Subsequently, we computed the linear prediction coefficients (LPC) for the signal by solving the Yule–Walker equations. The Yule–Walker equations are a set of linear equations used to compute the linear prediction coefficients (LPC) in the context of time-series analysis and signal processing. They are derived from the autocovariance function of a stationary stochastic process. Given a time series $x\left[n\right],$ the Yule–Walker equations for p order LPC can be written as

(3) R.a=r

where R is a Toeplitz matrix formed from the autocovariance function of the time series. The elements of R are given by

(4) $$R\left[i,j\right]=E\left[x\left[n-i\right.\right]\;\cdot x\left[n-\left.j\right]\right]$$

where E[] denotes the expected value. With the LPCs obtained, we proceeded to calculate the LPCCs using the following recursive relations:

(5) $$c_{j}=a_{j}+\sum_{t=1}^{j=1}\left(\frac{j-t}{j}\right)c_{t}a_{j-t},\;for\;1\leq j\leq p$$

(6) $$c_{j}=\sum_{t=1}^{j=1}\left(\frac{j-t}{j}\right)c_{t}a_{j-t},\;for\;p<j\leq d$$

**Figure 4:**
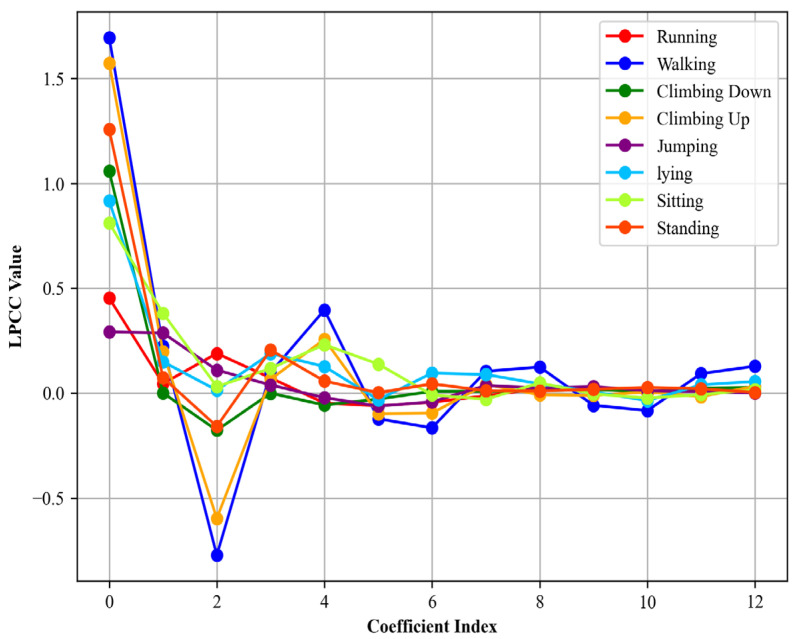
LPCCs computed for different locomotion activities over the Real-World Har dataset.

Here $c_{j}$ denotes the LPCCs, $a_{j}$ are the LPCCs, p is the order of the LPC model, and d represents the total number of LPCC coefficients to be computed. In Figure 4, the LPCCs were then plotted to offer a comprehensive view of the coefficient variations across different activities.

##### Dynamic Time Wrapping (DTW)

We utilized dynamic time warping (DTW) [44] to distinguish between different locomotion activities, e.g., running and walking activities, based on sensor data. We segmented the time-series data into overlapping windows and computed average patterns for running and walking as reference patterns, mathematically represented as

(7) $$active_{reference}=\frac{1}{n}\sum_{i=1}^{n}{running\_windows}_{i}$$

Similarly, for passive reference:

(8) $${passive}_{reference}=\frac{1}{m}\sum_{j=1}^{m}{walking\_windows}_{j}$$

For each window, we then calculated the DTW distance to both reference patterns, given by

(9) $$DTW\left(P,R\right)=min\sqrt{\sum_{k=1}^{K}p_{k}^{2}}\;$$

where P and R are the window and reference pattern, respectively.

**Figure 5:**
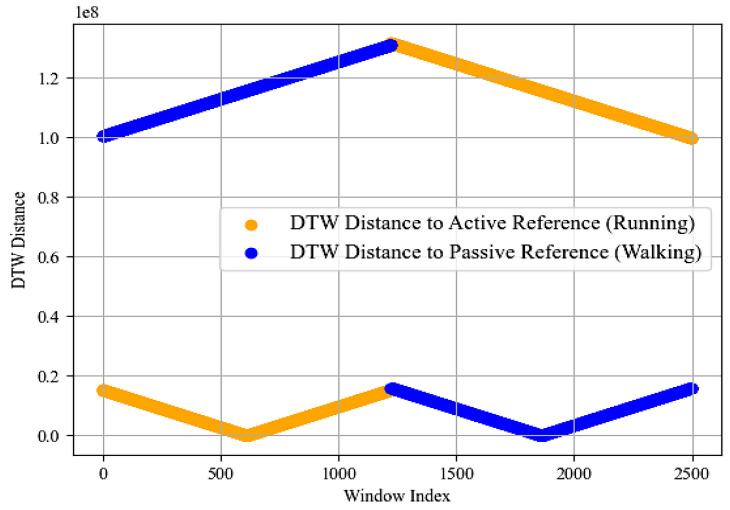
DTW was computed for different locomotion activities over the Real-World Har dataset.

DTW Distance to Active Reference refers to the distance between each window and the reference pattern for running, implying that smaller distances in this measure indicate similarity to running activity. DTW Distance to Passive Reference refers to the distance between each window and the reference pattern for walking, implying that smaller distances in this measure indicate similarity to walking activity. Figure 5 better summarizes the above discussion.

##### Spectrogram

We analyze the computational capabilities of spectrograms to examine patterns in microphone data, focusing on the auditory signatures of different human activities. The analysis began by segmenting the audio signal into overlapping frames and applying a window function, typically a Hamming window, as described in Section 3.2. The power spectrum of each frame was computed to elucidate the distribution of power across different frequencies. For the spectrogram analysis, the short-time Fourier transform (STFT) was employed to transform the audio signal into a time–frequency representation. The power spectrogram, *S(t, f)*, of the signal is formulated as

(10) $$S\left(t,f\right)={\left|F\left(t,f\right)\right|}^{2}$$

where *S*(*t*, *f*) represents the power of the frequency component *f* at time *t*, and *F(t*, *f)* is the STFT of the signal. This formulation allows for the visualization of the frequency spectrum over time, providing a detailed view of the acoustic properties of the activities being analyzed.

**Figure 6:**
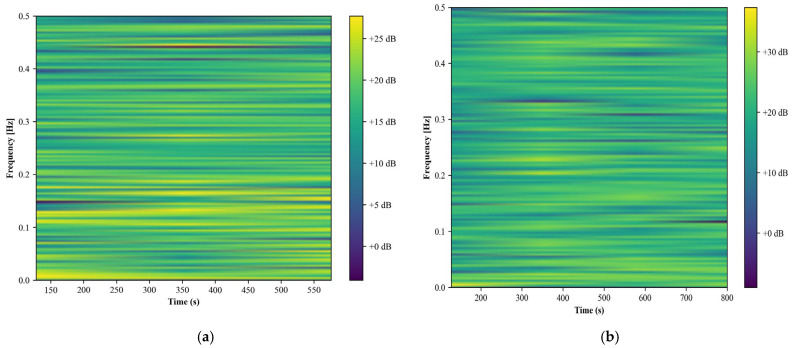
Spectrogram computed for (**a**) running and (**b**) walking over the Real-World HAR dataset.

In the walking activity spectrogram, we observe a more uniform distribution of frequencies with occasional spikes in intensity. These spikes may correspond to specific moments in the walking cycle, such as footfalls or contact with the ground. The overall intensity appears to be lower compared to the running activity, suggesting a softer acoustic profile. Conversely, the running activity spectrogram [45,46] displays a broader range of frequencies with more pronounced intensity variations. This pattern likely reflects the increased impact and movement associated with running, resulting in a richer and more varied acoustic signature. The higher intensities observed could be attributed to stronger footfalls and faster movements, which generate a wider array of sounds. Both spectrograms reveal the temporal evolution of the activities, with changes in sound profiles over time. In Figure 6, the walking spectrogram shows a more consistent pattern, whereas the running spectrogram exhibits greater variability and complexity. This difference underscores the distinct acoustic characteristics inherent to each activity.

##### State Space Correlation Entropy (SSCE)

State space correlation entropy (SSCE) [47,48,49] is a robust feature extraction method to quantify the variability and structure in the time-series data corresponding to each activity. The methodology commenced with the preprocessing of raw sensor data, calculated as the square root of the sum of the squares of the individual x, y, and z components. Subsequently, we applied an embedding technique to transform the scalar time-series data into a multidimensional state space. The embedding dimension, a critical parameter, was experimentally set to five to capture the intricate dynamics in the state space effectively. State space correlation entropy (SSCE) was computed based on the covariance matrix of the embedded vectors in the state space. The diagonal elements of the covariance matrix represented the variance of the embedded vectors, which were normalized to form a probability distribution. SSCE was then calculated as the negative sum of the product of the probabilities and the logarithm of the probabilities. Mathematically, for a given probability distribution $p=\left[p_{1,}p_{2}\ldots ..p_{n}\right]$:

(11) $$SSCE=-\sum_{i}^{n}p_{i}\log\left(p_{i}\right)$$

**Figure 7:**
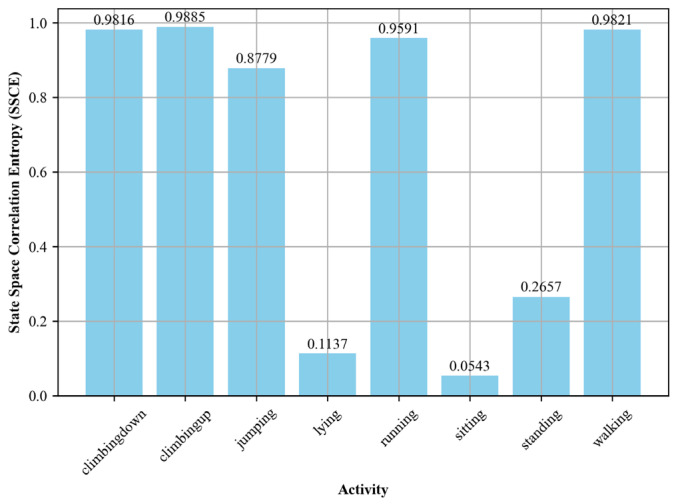
SSCE computed for different activities over the Real-World HAR dataset.

Figure 7 shows the SSCE values for each activity, visualized using bar plots.

##### Phase Angle

The phase angle is a valuable feature that provides useful information about the orientation and dynamics of body parts during different activities. It is particularly useful for distinguishing between activities that have similar magnitude of accelerations but differ in the spatial orientation of the movements. Given accelerometer data with readings in the *X* and *Y* directions, the phase angle *θ* for each time point is calculated using the arctangent function:

(12) $$\theta \left(t\right)=arctan\left(\frac{y(t)}{x(t)}\right)$$

Here, x(t) and y(t) are sensor readings in *X* and *Y* directions in time *t*. The arctangent function is used to compute the angle whose tangent is the quotient of the two specified numbers, yielding the phase angle in radians. This angle [50] is then converted to degrees for better interpretability:

(13) $$\theta_{degress}\left(t\right)=\theta \left(t\right)\times \left(\frac{180}{\pi }\right)$$

**Figure 8:**
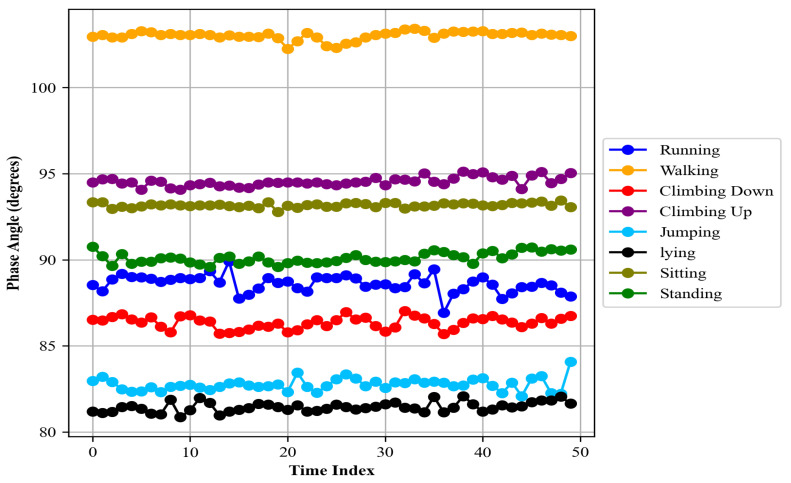
Phase angle calculated for different activities over the Real-World HAR dataset.

The resulting phase angles for each activity can be seen in Figure 8.

##### Skewness and Kurtosis

In this step, we extracted skewness and kurtosis [51,52] for locomotion data. Skewness quantifies the asymmetry of a probability distribution. A distribution with skewness equal to zero is perfectly symmetric, while a positive skewness indicates a distribution that is skewed or tailed on the right, and a negative skewness signifies a distribution that is skewed or tailed on the left. Mathematically, the skewness of a sample is calculated as

(14) $$Skewness=\frac{n\sqrt{n-1}}{n-2}\frac{\sum_{i=1}^{n}{\left(x_{i}-\bar{x}\right)}^{3}}{s^{3}}$$

where n is the sample size, $x_{i}$ are the sample observations, $x_{i}$ is the sample mean, and *s* is the sample standard deviation. Kurtosis [53], on the other hand, measures the tailedness of a probability distribution. It describes the height and sharpness of the central peak relative to that of a standard bell curve. A distribution with kurtosis [54] equal to three is considered normal. Excess kurtosis is often used, which is calculated by subtracting three from the kurtosis, to compare with the normal distribution. Distributions with high kurtosis have heavy tails and outliers, while those with low kurtosis are light-tailed with fewer outliers. The formula for calculating sample kurtosis is

(15) $$Kurtosis=\frac{n(n+1)}{\left(n-1)(n-2)(n-3\right)}\sum_{i=1}^{n}{\left(\frac{x_{i}-\bar{x}}{s}\right)}^{4}-\frac{{3(n-1)}^{2}}{\left(n-2)(n-3\right)}$$

**Figure 9:**
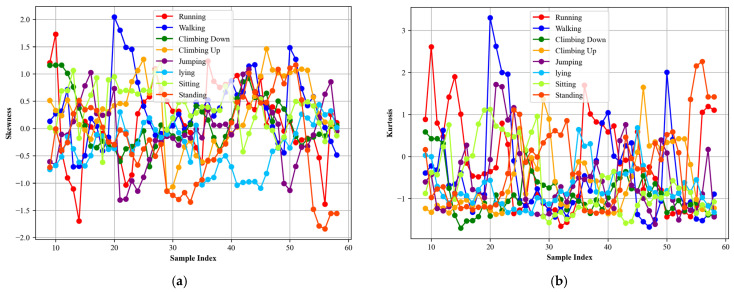
Skewness and kurtosis calculated for different activities over the Real-World HAR dataset, (**a**) Skewness (**b**) Kurtosis.

Figure 9 presents skewness and kurtosis for different activities.

#### 3.3.2. Feature Extraction for Localization Activity

In this part, we extract different features for localization activities [55,56,57,58]. Each feature is described in detail below.

##### Step Count Detection

In our approach to human localization, we emphasize the importance of step detection as a foundational element in pedestrian dead reckoning (PDR) systems. To accurately quantify the steps taken [59], we first analyze accelerometer data to capture the intricate motion patterns associated with human activity. We then compute the magnitude of acceleration by consolidating the triaxial accelerometer readings $\left(b_{x,}b_{y,}b_{z}\right)$ into a singular metric using the equation

(16) $$M=\sqrt{{b_{x}}^{2}+{b_{y}}^{2}+{b_{z}}^{2}}$$

**Figure 10:**
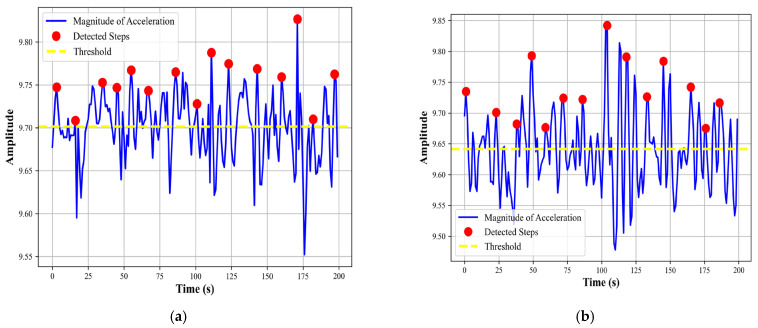
Step count detected for (**a**) walking and (**b**) running activities over the Real-World HAR dataset.

We employ a peak detection algorithm on *S* selecting peaks that surpass a certain threshold *T* defined as a proportion of the signal’s amplitude and are separated by at least a minimum distance *D* reflective of expected gait cadence. This methodical approach enables us to detect individual steps and accumulate a total step count, which is crucial for path reconstruction in GPS-denied environments. The step count detected for walking and running activity can be seen in Figure 10.

##### Heading Angle

The calculation of the absolute heading angle is integral to our localization framework. We utilize magnetometer data to determine orientation concerning the Earth’s geomagnetic field. By computing the arctangent of the magnetometer’s *y*-axis $\left(a_{y}\right)$ over its *x*-axis $\left(a_{x}\right)$ readings, we derive the raw heading [60,61] in degrees:

(17) $$H_{rad}=arctan2\left(a_{y},a_{x}\right)$$

The value is then converted into degrees and normalized to ensure a range from 0° to 360°, yielding the absolute heading angle

(18) $$H_{deg}=\left(H_{rad}\cdot \frac{180}{\pi }\right)mod\;360$$

**Figure 11:**
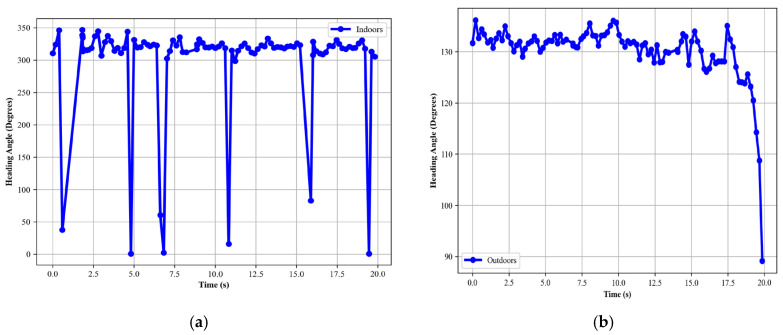
Heading angle calculated for (**a**) indoor and (**b**) outdoor activity over the Extrasensory dataset.

The heading angle calculated for indoor and outdoor is shown in Figure 11.

##### Skewness and Kurtosis for Localization Activity

**Figure 12:**
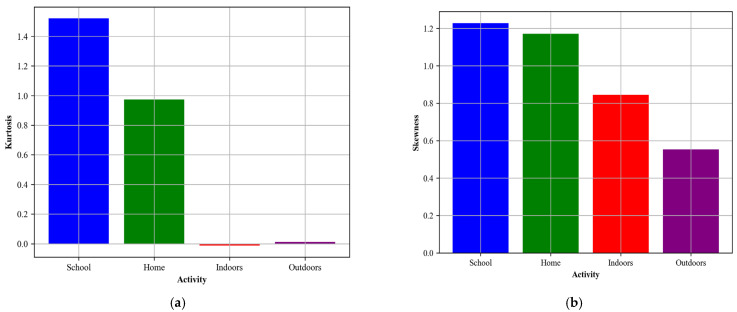
Skewness and kurtosis were calculated for different activities over the Extrasensory dataset, (**a**) Kurtosis (**b**) Skewness.

We separately extract skewness and kurtosis features for localization activity. We already have discussed these in detail about these features in (Section 3.3.1). In Figure 12, the visualizations can be seen.

### 3.4. Feature Selection Using Recursive Feature Elimination

**Figure 13:**
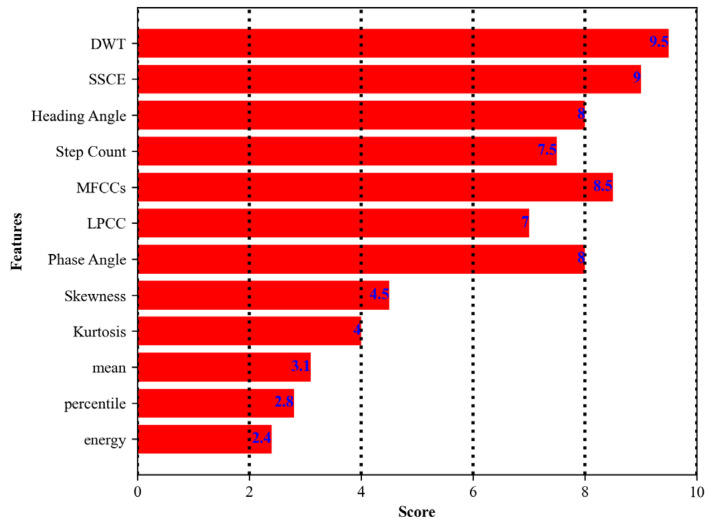
Score for individual feature using recursive feature elimination.

Recursive feature elimination (RFE) [60,61,62] is a feature selection method that aims to choose the most significant features in a given model. It is particularly useful when dealing with a high-dimensional dataset where feature selection becomes crucial. We extracted different features for locomotion and localization activities. We initiated the process with a set of all available features. Every feature in this set had the potential to contribute to the model’s predictive power, but the goal was to include which ones were the most influential. Then, the model is trained on all available features. Post-model training, we ranked the features based on their importance, derived from the model’s coefficients or feature importance. The significance of each feature was evaluated in the context of its contribution to the model’s output. We identified and eliminated the least significant feature from the feature set. This step was important as it refined the feature set by removing features that were not contributing substantially to the model’s predictive power. In this way, we reduced the feature set. It is important to note that all the features that are discussed above are selected for training the model; rejected features include, mean, percentile, and energy. The algorithm working of recursive feature elimination [63] is shown in Algorithm 1. The plot for feature importance can be seen in Figure 13.
**Algorithm 1.** Recursive Feature Elimination1: **Input**: D: Dataset with N instances and M features. 2: model: Predictive model to be used. 3: k: Desired number of features to select. 4: **Output**: 5: $F_{selected}$: Selected feature subset of size *k*. 6: ${model}_{performance}:\;$Performance of the model with selected features. **7:** Initialization: 8: Train the model with all *M* features in the dataset *D*. 9: Compute the initial ${model}_{performance}$ **10:** **Feature Selection and Elimination:** 11: **for** i = 1 to M-k do 12: - Rank the features based on their importance as determined by the model. 13: - Identify the least important feature (f_{least}). 14: - Remove (f_{least}) from the dataset (D). 15: - Re-train the (model) with the remaining features in (D). 16: - Update (model_{performance}) with the new model. 17: **end for** **18:** **Selection:** 19: The remaining *k* features in *D* are the selected features $F_{selected}$. **20:** **Return:** 21: return the selected feature subset $F_{selected}$ and the final ${model}_{performance}$ **22:** **End**

### 3.5. Data Augmentation

In our study, we incorporated the synthetic minority oversampling technique (SMOTE) [64] to address the issue of class imbalance prevalent in our dataset. The presence of imbalanced classes can lead to a biased model that tends to favor the majority class, thereby undermining the model’s predictive accuracy for the minority class. To mitigate this, we opted for SMOTE, which aims to balance the class distribution by generating synthetic instances of the minority class. We observed that the Extrasensory dataset has an imbalanced nature for certain activities; notably, at school and outdoors, SMOTE was applied to minority classes (At_School, Or_outside). Before applying SMOTE, we observed poor performance of the model, resulting in low accuracy. After applying SMOTE, we obtained a very good improvement in model performance. The count of the number of samples for each class is presented in Table 2 and Table 3.

### 3.6. Activity Recognition

**Figure 14:**
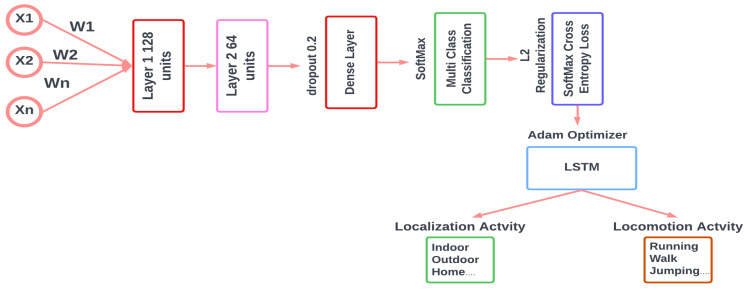
Proposed LSTM architecture.

We initiated the neural network’s architecture by defining specific weights and biases. The architecture prominently features a long short-term memory (LSTM) model, a specialized form of recurrent neural network (RNN), good for sequence and time-series data. This LSTM model processes the input data, and takes advantage of the predefined weights and biases, to generate predictive outcomes. To ensure the robustness of our model and mitigate overfitting, especially given the potential complexity of the neural network, we incorporated L2 regularization. This technique introduces a penalty to the loss function based on the magnitude of the weights, thereby encouraging the model to maintain smaller weight values. For the loss function, we used softmax cross-entropy, which quantifies the prediction error, with the L2 loss, ensuring a balance between data fitting and overfitting prevention. For the optimization phase, we employed the Adam optimizer. This optimizer dynamically adjusts the learning rate throughout the training process, often leading to expedited convergence and enhanced model generalization. The architecture of the proposed LSTM is shown in Figure 14.

## 4. Experimental Setup

The experimental segment is further broken down into distinct subsections. Initially, we detail the dataset employed in our research. Following that, we showcase the accuracy of each activity through a confusion matrix. Subsequently, we compute the precision, recall, and F1 scores for various activities. In the subsequent phase, we plot the ROC curve for all activities. Afterward, we provide a comparison with other classifiers. Finally, we conclude and discuss future directions.

### 4.1. Datasets Descriptions

In this section, we delve into the specifics of each dataset, highlighting their diversity and how they reflect real-world scenarios.

#### 4.1.1. The Real-World HAR Dataset

The Real-World Human Activity Recognition (HAR) dataset is a rich collection of sensor data [65] that reflects a broad spectrum of physical human activities, making it an invaluable resource for understanding and analyzing human motion in diverse settings. This dataset encompasses data from 15 participants who were engaged in eight different locomotion activities, including walking, running, lying, climbing up and down, standing, jumping, and sitting. What makes this dataset particularly relevant to real-world scenarios is the variety of sensor placements on the participants’ bodies. Sensors such as accelerometers, gyroscopes, magnetometers, GPS, light, and microphones were strategically positioned on various body parts chest, head, forearm, shin, thigh, upper arm, and waist. This diverse sensor placement captures a comprehensive range of motion dynamics and patterns, reflecting the complex and varied nature of human activities in everyday life. The Real-World HAR dataset is thus instrumental in developing systems that require an understanding of human movements in different physical contexts, making it ideal for applications in areas such as fitness tracking, health monitoring, and ergonomic studies. All the activities are mentioned in Table 4.

#### 4.1.2. The Real-Life HAR Dataset

The Real-Life HAR dataset [66] stands out for its capture of human activity in natural, uncontrolled environments, providing a window into the spontaneous and everyday interactions of individuals with their devices. Collected from 19 individuals using an Android application, this dataset includes a variety of common activities categorized as ‘Inactive’ (when the smartphone is not on the person), ‘Active’ (general movement with the phone but not traveling), ‘Walking’ (purposeful movement towards a destination), and ‘Driving’ (movement within a vehicle). The unstructured nature of this data collection mimics the randomness and unpredictability of daily life, making it a valuable asset for testing and enhancing activity recognition systems in real-world scenarios. The Real-Life HAR dataset challenges these systems to accurately identify and classify a wide range of activities that occur in typical day-to-day situations, from sedentary behaviors like sitting at a desk to active behaviors like walking or driving. It is particularly beneficial for developing smart device applications aimed at lifestyle monitoring, urban mobility analysis, and personal safety.

#### 4.1.3. The Extrasensory Dataset

The Extrasensory dataset is a comprehensive and multidimensional collection of sensor data that captures a wide array of human activities, ranging from basic movements to complex behaviors [67]. Data were collected from various smartphones and smartwatches, incorporating sensors such as accelerometers, gyroscopes, magnetometers, and GPS. This dataset stands out for its inclusivity of not only fundamental activities like walking or running but also intricate behaviors like cooking, shopping, or engaging in social activities. The diverse nature of the data collection, encompassing different sensor types and activities, makes the Extrasensory dataset particularly reflective of the multifaceted nature of human behavior in various environments. Participants in the study were monitored over extended periods in their natural environments, ensuring that the data capture the genuine and spontaneous nature of human actions. This real-world aspect of the dataset makes it invaluable for developing robust activity recognition models that can adapt to and accurately predict human behavior in a wide range of contexts. Applications leveraging the Extrasensory dataset are well-suited for personalized and context-aware services in fields like health monitoring, fitness tracking, smart home automation, and ambient assisted living.

### 4.2. Confusion Matrix

We have illustrated the confusion matrix for each activity. We have provided a thorough and detailed discussion of the interpretations drawn from each confusion matrix. The confusion matrix was constructed by comparing the predicted labels against the true labels from the test set.

### 4.3. Precision, Recall, and F1 Score Computation

In this study, precision, recall, and F1 scores for various activities across different datasets were presented, showcasing the performance of the model on the Real-World HAR, Real-Life HAR, and Extrasensory datasets. The precision metric reflects the model’s accuracy in predicting identifications, recall measures the model’s ability to detect all actual positives, and the F1 score provides a harmonic mean of precision and recall, offering a balance between the two.

### 4.4. Receiver Operating Characteristic (ROC)

We also computed the receiver operating characteristic (ROC) curve [68], a graphical representation that illustrates the diagnostic ability of our classification system. This curve is created by plotting the true positive rate (TPR) against the false positive rate (FPR) at various threshold settings. The TPR is also known as sensitivity, recall, or probability of detection, and it measures the proportion of actual positives that are correctly identified. The FPR, on the other hand, is the probability of false alarm, and represents the proportion of actual negatives that are incorrectly identified as positive. The area under the ROC curve (AUC) provides a measure of the model’s ability to distinguish between the classes. An AUC of 1.0 indicates perfect classification, while an AUC of 0.5 implies that the model is no better than random guessing. In this study, we calculated the ROC curve for each class in our multiclass classification problem. By analyzing the shape and area under these curves, we were able to assess the performance of our classification model in distinguishing between the different activities.

## 5. Results and Discussion

In this section, we presented the results obtained for different datasets in detail.

### 5.1. First Experiment: Confusion Matrix Results

We present the confusion matrix results for the Real-World HAR, Real-Life Har, and Extrasensory datasets and discuss the model’s performance for each activity. The results obtained can be seen in Table 5, Table 6 and Table 7.

#### 5.1.1. Discussion and Analysis of Real-World Har Dataset (Confusion Matrix)

The confusion matrix for the Real-World HAR dataset in Table 4, demonstrates that the model performs exceptionally well for ‘running’, ‘walking’, ‘lying’, and ‘standing’, with respective accuracies of 98%, 96%, 100%, and 98%. This indicates that the model is highly capable of distinguishing these activities, which is critical in applications such as fitness tracking and patient monitoring systems. However, activities like ‘climbing up’ and ‘jumping’ have lower true positive rates, indicating potential challenges in distinguishing these activities from others like ‘climbing down’ or ‘sitting’. This could be due to similar sensor patterns between these activities. In practical scenarios like emergency response or activity-based user interface control, this misclassification could lead to unintended actions. The mean accuracy of 85.73% reflects an overall strong performance but also highlights the need for further model refinement for certain activities.

#### 5.1.2. Discussion and Analysis of Real-Life Har Dataset (Confusion Matrix)

In Table 5, ‘active’ and ‘walking’ activities are well recognized, with accuracies of 83% and 88%, respectively, which is important for applications such as automated activity tracking in smartphones. ‘Inactive’ and ‘driving’ also show high recognition rates of 96% and 87%, which can be essential for vehicle safety systems to detect driver activity. The overall mean accuracy of 89.53% shows a good model performance in a real-life setting. The confusion between ‘active’ and other classes such as ‘inactive’ and ‘driving’ can be a limitation, possibly due to overlapping sensor signatures during low-intensity active periods or active driving. Understanding this is important for applications where differentiating between subtle movements is crucial.

#### 5.1.3. Discussion and Analysis of Extrasensory Dataset (Confusion Matrix)

Finally, in Table 6, the confusion matrix for the Extrasensory dataset shows high accuracy across all classes, with ‘Loc_home’, ‘Indoors’, and ‘Outdoors’ above 87%. This is significant for location-based services and smart home systems, where accurate context recognition is necessary to provide personalized experiences. The model shows a slight confusion between ‘At_School’ and ‘Loc_home’, potentially due to the similarity in stationary patterns while at these locations. This indicates a limitation in distinguishing between stationary contexts, which can be critical in applications such as energy-saving systems where precise location context is required. The high mean accuracy of 95.21% is promising for robust location-aware applications.

### 5.2. Second Experiment: Precision, Recall, and F1 Score Results

In this section, a detailed discussion on the observed precision, recall, and F1 scores for the Real-World HAR, Real-Life HAR, and Extrasensory datasets, noting the high performance for certain activities and the need for improvement in others. In Table 8, the performance metrics for the proposed system are presented.

#### 5.2.1. Discussion and Analysis of Real-World Har Dataset (Precision, Recall, and F1 Score)

For Real-World HAR, the model exhibits varied precision across different activities. For ‘lying’, the precision is nearly perfect, indicating that when the model predicts this activity, it is almost always correct. This precision is crucial for health-monitoring applications where false positives for rest could lead to incorrect health status assessments. On the other hand, ‘jumping’ has a lower precision, which might affect applications that rely on detecting specific motion patterns, such as sports analytics. The recall scores highlight the model’s ability to correctly identify true instances of each activity. High recall for ‘running’ and ‘standing’ is essential for reliable activity tracking. However, the lower recall for ‘climbing up’ might lead to underdetection in scenarios like stair-climbing exercises where each step counts. The F1-scores, which combine precision and recall, suggest that the model is most effective at recognizing ‘lying’, ‘running’, and ‘standing’. This efficacy is beneficial for applications that need to differentiate between sedentary and active states. However, the lower F1 scores for ‘climbing down’ and ‘jumping’ suggest that the model’s performance could be improved for applications requiring fine-grained activity recognition, such as detailed fitness tracking or advanced user interface interactions.

#### 5.2.2. Discussion and Analysis of Real Life Har Dataset (Precision, Recall, and F1 Score)

For Real-Life HAR, precision is high for ‘inactive’ and ‘driving’ activities, which is significant for applications like automated activity logs in vehicles or smart devices. The model’s high recall for ‘inactive’ suggests that it is adept at capturing periods of inactivity, which is vital for sedentary behavior research. The F1 scores show the model’s balanced performance for detecting ‘active’ versus ‘inactive’ states, which can inform systems designed to encourage a more active lifestyle by nudging users at the right time.

#### 5.2.3. Discussion and Analysis of Extrasensory Dataset (Precision, Recall, and F1 Score)

For the Extrasensory dataset, the high precision for ‘At_School’ and ‘Indoors’ indicates the model’s reliability in these contexts, which can enhance educational and indoor navigation applications. The recall for ‘Loc_home’ and ‘Outdoors’ activities is commendable, ensuring that the system reliably detects when a user is at home or outdoors, enabling applications like smart home systems and outdoor fitness apps to function accurately. The impressive F1 scores across all locations demonstrate the model’s robustness in location-aware contexts, laying a foundation for precise context-aware services that can adapt to the user’s current environment.

### 5.3. Third Experiment: ROC Curve Results

**Figure 15:**
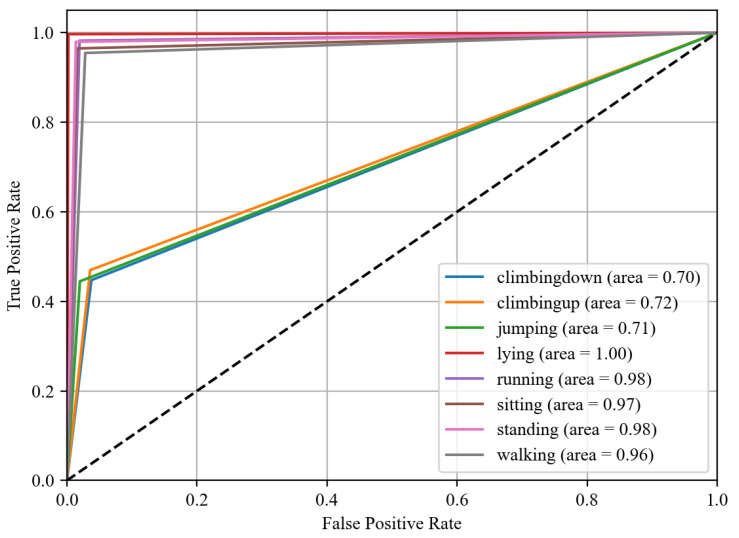
ROC curve for Real-World Har dataset.

**Figure 16:**
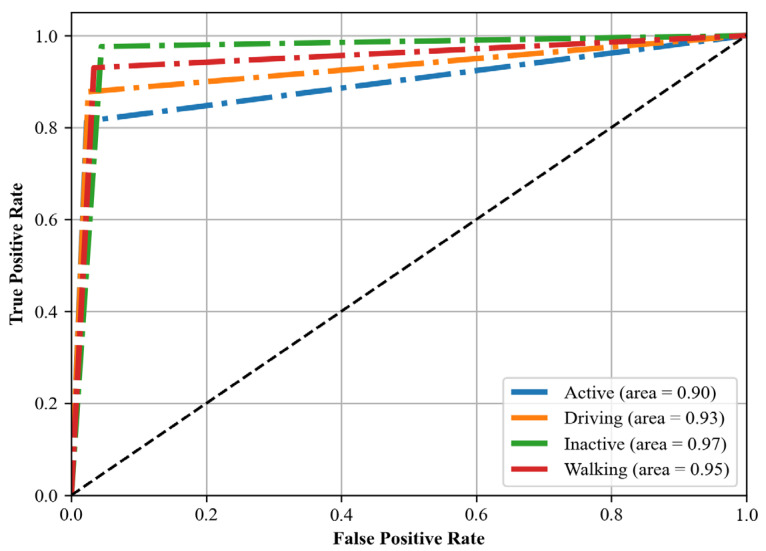
ROC curve for Real-life HAR dataset.

**Figure 17:**
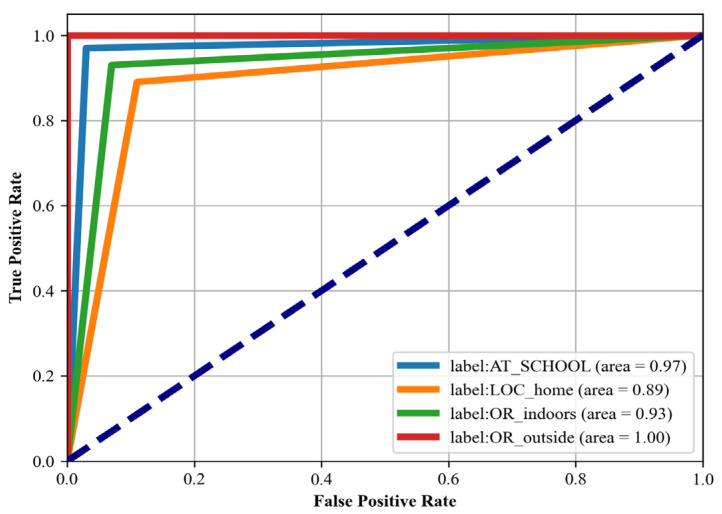
ROC curve for Extrasensory dataset.

In this section, the implications of the ROC curve analysis are discussed, highlighting the model’s discriminative capabilities across the various datasets. Figure 15, Figure 16 and Figure 17 show the roc curve for Real-World HAR, Real-Life HAR, and Extrasensory datasets.

#### 5.3.1. Discussion and Analysis of Real-World Har Dataset (Roc Curve)

For the Real-World HAR dataset, the model excels at distinguishing ‘lying’, ‘running’, ‘sitting’, and ‘standing’ activities with ROC areas close to 1.00, indicating almost perfect classification capabilities; it faces more significant challenges with activities such as ‘climbing up’, ‘climbing down’, and ‘jumping’, where the areas are in the 0.70 range. These activities, characterized by complex movement patterns and potential similarities in sensor data, require more sophisticated modeling to improve recognition. Nevertheless, the high ROC areas for the majority of activities reflect the robust ability of the model to correctly classify common activities, which is critical for a variety of applications including fitness tracking, patient monitoring, and personal safety devices.

#### 5.3.2. Analysis of Real-Life Har Dataset (Roc Curve)

The Real-Life HAR dataset’s ROC curve analysis shows the model’s strong performance in classifying ‘active’, ‘inactive’, ‘driving’, and ‘walking’ states with high confidence, as indicated by ROC areas above 0.90. The model’s ability to accurately identify these states is particularly beneficial for applications that aim to promote health and safety, such as activity tracking in wearable devices and driver alertness monitoring systems. The high discrimination power for ‘inactive’ and ‘driving’ states, with areas nearing 0.97 and 0.93, respectively, underscores the model’s potential in applications that require precise detection of sedentary behavior or driving activities. Meanwhile, the slightly lower but still robust area for ‘active’ states points to the model’s capability to capture a wide range of general activities, which is crucial for comprehensive activity monitoring systems.

#### 5.3.3. Analysis of Extrasensory Dataset (Roc Curve)

For the Extrasensory HAR dataset, the model demonstrates outstanding classification performance across different location contexts, with ROC areas indicating a high level of discrimination for ‘Loc_home’, ‘Indoors’, and ‘Outdoors’. The perfect ROC area for ‘Outdoors’ activities showcases the model’s exceptional capability to identify outdoor contexts, which could be leveraged in outdoor navigation and fitness applications. The high area for ‘At_School’ activities suggests that the model can also effectively differentiate academic-related contexts, offering possibilities for applications in educational settings. Although the area for ‘Loc_home’ is slightly lower, it still represents a fairly good performance in recognizing home-related activities, which is vital for smart home automation and energy management systems. The overall excellent ROC areas across all classes affirm the model’s utility in providing accurate context-aware services in a wide range of environments.

#### 5.3.4. Fourth Experiment: Comparison with Other Techniques

In the last experiment, the proposed system is compared with the state-of-the-art techniques. In [69], researchers utilized a random forest algorithm to analyze the Real-World HAR dataset, applying the model in two distinct operational scenarios. The first scenario assumed knowledge of the sensor’s placement on the participant’s body, while the second did not. Without information on the sensor’s position, the model achieved an accuracy of 80.2%. However, when the sensor placement was considered, the accuracy improved to 83.4%, indicating that awareness of device location can enhance the predictive performance of human activity recognition systems. In reference [70], researchers employed a cross-subject activity recognition model, which yielded an accuracy rate of 83.1%. Meanwhile, the study cited as [71] utilized signal visualization techniques in conjunction with a convolutional neural network (CNN), achieving an impressive 92.4% accuracy on the Real-World HAR dataset. In the study mentioned in [72], the authors applied a support vector machine (SVM) to analyze the efficacy of various sensor combinations. They started with an accelerometer and GPS, reaching an accuracy of 60.1%. When they included a magnetometer alongside the initial sensors, the accuracy saw an uptick to 62.6%. Furthermore, by integrating gyroscope data into the mix, they were able to enhance the accuracy to 67.2% (Table 9).

## 6. Implications of the Proposed System

In the realm of wearable technology, the practical application of our proposed system extends far beyond the confines of a controlled research environment, demonstrating significant potential in a variety of real-world settings. Envisioned primarily for health monitoring and fitness tracking, the system offers accurate and continuous assessment of physical activities, aiding in personalized health management and wellness monitoring. Its applicability in sports science is particularly noteworthy, where it can provide athletes and coaches with detailed insights into training regimes and performance metrics. Furthermore, the system’s ability to recognize and differentiate between a myriad of human activities positions it as an invaluable tool in elderly care and assisted living environments, where accurate activity monitoring can enhance the safety and well-being of individuals. In urban settings, the integration of our system into smart city frameworks could revolutionize how daily activities are monitored and analyzed, contributing to smarter health and lifestyle choices among urban dwellers. Additionally, its use in occupational health, particularly in monitoring workplace ergonomics, can help in mitigating work-related injuries and enhancing productivity. The diversity of the datasets used ensures that the system is well-adapted to various environments, from indoor settings to outdoor scenarios, making it a versatile solution for myriad applications. This broad spectrum of applicability underscores the system’s potential to significantly impact various facets of modern life, where the integration of smart wearable technology is increasingly becoming indispensable.

## 7. Conclusions, Limitations, and Future Work

Our research has successfully developed a hybrid LSTM-based system for recognizing human locomotion and localization activities using data from smartphone and smartwatch sensors. The choice of LSTM, owing to its proficiency in handling time-series data, proved effective, as evidenced by our system achieving accuracies of 89% for Real-Life HAR, 85% for Real-World HAR, and 95% for the Extrasensory dataset. These results not only demonstrate an advancement over existing methods but also highlight our system’s capability in processing diverse and complex sensor data streams.

We recognize certain limitations in our study, particularly concerning the variability in human activities and the challenges it presents in achieving high precision and recall for less common activities. The system’s current reliance on specific datasets and the preprocessing steps may also limit its generalizability.

To address these limitations and further enhance our system, our future research will focus on several key areas.

- Expanding Dataset Diversity: We plan to test and refine our system with a broader range of datasets, especially those with higher-dimensional data and less distinguishable activity patterns, to improve its adaptability and robustness in varied real-world scenarios.
- Advanced Oversampling Techniques: Investigating more sophisticated oversampling methods and deep learning architectures will be a priority, aiming to improve the handling of imbalanced datasets.
- Exploring Federated Learning: We intend to explore the potential of federated learning to enhance model robustness and privacy, which could significantly benefit the deployment of our system in privacy-sensitive applications.
- Environmental Impact Analysis: Understanding the impact of dynamic environmental factors on sensor data quality and system performance will be crucial. We will conduct studies to evaluate how varying environmental conditions affect the accuracy and reliability of our system.
- User-Centered Design: To improve system acceptability and usability, we will focus on user-centered design principles, ensuring the user interface is intuitive, accessible, and tailored to the needs and preferences of diverse user groups.
- Addressing Privacy Concerns: We will prioritize the implementation of robust data privacy measures, including data anonymization and secure data processing, to ensure user trust and compliance with data protection regulations.

## Figures and Tables

**Table 1:** Feature list along with sensor type.

Feature	Sensor Type
Statistical features (temporal domain)	IMU sensors
2. Spatial/geometric features	IMU sensors
3. Spectral features	IMU, audio sensors
4. Entropy features	IMU sensors
5. Distance measures	IMU and GPS sensor

**Table 2:** Original instance counts for each class in the Extrasensory dataset.

Class Label	Instances
OR_indoors	184,692
2. At_School (minority class)	42,331
3. Loc_Home:	152,892
4. Or_outside (minority class)	12,114

**Table 3:** SMOTE before and after precision, recall, and F1 score over Extrasensory dataset.

Class Label	Before SMOTE			After SMOTE		
	Precision	Recall	F1 Score	Precision	Recall	F1 Score
OR_indoors	0.81	0.89	0.93	0.95	0.98	1.00
2. At_School	0.75	0.71	0.66	0.91	0.90	0.92
3. Loc_Home	0.88	0.88	0.85	0.90	1.00	0.95
4. Or_outside	0.61	0.72	0.65	0.90	1.00	0.88

**Table 4:** Human locomotion activities provided by Real-World HAR.

Serial No	Real-World HAR
1.	Walking
2.	Running
3.	Lying
4.	Climbing up
5.	Climbing down
6.	Standing
7.	Jumping
8.	Sitting

**Table 5:** Confusion matrix over Real-World HAR dataset.

Obj. Classes	Clmup	Clmdwn	Run	Walk	Lying	Stnd	Jmp	Sit
**Clmup**	**0.65**	0.09	0.05	0.04	0.00	0.04	0.05	0.08
clmdwn	0.06	**0.72**	0.04	0.00	0.08	0.03	0.00	0.07
run	0.00	0.00	**0.98**	0.02	0.00	0.00	0.00	0.00
walk	0.00	0.00	0.00	**0.96**	0.00	0.04	0.00	0.00
lying	0.00	0.00	0.00	0.00	**1.00**	0.00	0.00	0.00
stnd	0.00	0.00	0.00	0.00	0.00	**0.98**	0.02	0.00
jmp	0.03	0.02	0.09	0.00	0.00	0.08	**0.64**	0.14
sit	0.00	0.03	0.00	0.00	0.00	0.00	0.00	**0.97**
**Mean Accuracy = 85.73%**								

clmp = climbing up; clmdwn = climbing down; run = running; stnd = standing; jmp = jumping; sit = sitting.

**Table 6:** Confusion matrix over Real-Life HAR dataset.

Obj. Classes	Active	Inactive	Driving	Walking
Active	**0.83**	0.09	0.04	0.04
Inactive	0.00	**0.96**	0.04	0.00
Driving	0.05	0.06	**0.87**	0.02
Walking	0.02	0.07	0.03	**0.88**
**Mean Accuracy = 89.53%**				

**Table 7:** Confusion matrix over Extrasensory dataset.

Obj. Classes	At_School	Loc_Home	Indoors	Outdoors
At_School	**0.83**	0.09	0.05	0.04
Loc_home	0.06	**0.96**	0.04	0.00
Indoors	0.00	0.00	**0.87**	0.02
Outdoors	0.00	0.00	0.00	**0.88**
**Mean Accuracy = 95.21%**				

**Table 8:** Classification report for Real-World Har, Real-Life Har, and Extrasensory Datasets.

Classes	Real-World Har			Real-Life Har				Extrasensory	
Activities	Precision	Recall	F1 Score	Precision	Recall	F1 Score	Precision	Recall	F1 Score
Climbing Down	0.58	0.45	0.51	-	-	-	-	-	-
Climbing Up	0.63	0.48	0.55	-	-	-	-	-	-
Jumping	0.53	0.40	0.42	-	-	-	-	-	-
Lying	0.99	1.00	1.00	-	-	-	-	-	-
Running	0.88	0.98	0.99	-	-	-	-	-	-
Sitting	0.92	0.97	0.93	-	-	-	-	-	-
Standing	0.92	0.98	0.95	-	-	-	-	-	-
Walking	0.83	0.96	0.89	-	-	-	-	-	-
Inactive	-	-	-	0.98	0.96	0.97	-	-	-
Active	-	-	-	0.75	0.83	0.90	-	-	-
Walking	-	-	-	0.93	0.88	0.90	-	-	-
Driving	-	-	-	0.90	0.88	0.92	-	-	-
At_School	-	-	-	-	-	-	0.98	0.99	0.94
At_Home	-	-	-	-	-	-	0.99	0.90	0.87
Indoors	-	-	-	-	-	-	0.95	0.93	1.00
Outdoors	-	-	-	-	-	-	1.00	0.95	0.92
**Mean**	0.79	0.78	0.80	0.86	0.88	0.87	0.97	0.94	0.93

**Table 9:** Comparison of proposed hybrid LSTM with other methods.

Method		Accuracy %	
	Real-World HAR	Real-Life HAR	Extrasensory
T. Sztyler et al. [69]	80.2	-	-
S. Korakot et al. [70]	72.8	-	-
Stuckenschmidt et al. [71]	83.1	-	-
Luaces et al. [71]	-	60.21	-
Luaces al. [71]	-	62.60	-
Luaces et al. [71]	-	67.23	-
Mekruksavanich et al. [72]	-	70.3	
Vaizman et al. [73]	-	-	0.83
Asim et al. [74]	-	-	0.87
Abdullah et al. [75]	-	-	0.87
Usman et al. [76]		-	0.93
**Proposed**	**85.72**	**87.43**	**0.95**

## Data Availability

No new data were created or analyzed in this study. Data sharing is not applicable to this article.
